# Effectiveness and safety of asynchronous telemedicine consultations in general practice: a systematic review

**DOI:** 10.3399/BJGPO.2023.0177

**Published:** 2024-02-07

**Authors:** Cara Leighton, Alison Cooper, Annavittoria Porter, Adrian Edwards, Natalie Joseph-Williams

**Affiliations:** 1 Cardiff University School of Medicine, Cardiff, UK; 2 Division of Population Medicine Cardiff University, Cardiff, UK

**Keywords:** general practice, telemedicine, quality of health care, primary healthcare, general practitioners

## Abstract

**Background:**

There is a focus on increasing asynchronous telemedicine use, which allows medical data to be transmitted, stored, and interpreted later; however, limited evidence of the quality of care it allows in general practice hinders its use.

**Aim:**

To investigate uses and effectiveness of asynchronous telemedicine in general practice, according to the domains of healthcare quality, and describe how the COVID-19 pandemic changed its use.

**Design & setting:**

Systematic review in general practice.

**Method:**

A systematic search was carried out across four databases using terms related to general practice, asynchronous telemedicine, uses, and effectiveness, and supported by citation searching. This was followed by screening according to pre-defined criteria, data extraction, and critical appraisal. Narrative synthesis was then undertaken guided by the six domains of healthcare quality and exploring differences in use before and following the COVID-19 pandemic.

**Results:**

Searches yielded 6864 reports; 27 reports from 23 studies were included. Asynchronous telemedicine is used by a range of staff and patients across many countries. Safety and equity are poorly reported but there were no major safety concerns. Evidence from other domains of healthcare quality show effectiveness in making diagnoses, prescribing medications, replacing other consultations, providing timely care, and increased convenience for patients. Efficiency is impacted by negative effects on workflow, through poor implementation and patient non-adherence, limiting usability and requiring new administrative approaches from healthcare staff. Asynchronous telemedicine use increased rapidly from March 2020, following the COVID-19 pandemic outbreak.

**Conclusion:**

Asynchronous telemedicine provides quality care for patients but is limited by reports of increased workload and inefficient workflow compared with face-to-face consultations. Limits of evidence include heterogeneity and small-scale studies. Further research into cost-effectiveness, equity, safety, and sustained implementation will influence future policy and practice.

## How this fits in

Asynchronous telemedicine utilisation increased in general practice following the COVID-19 pandemic outbreak as approaches to replace face-to-face consultations were required for safety and infection control reasons, but there is little evidence of its effectiveness and safety. This review found asynchronous telemedicine can be effective for making diagnoses, prescribing medications, and providing timely care and increased convenience for patients. It takes equivalent time to face-to-face and telephone consultations, but is limited by reports of increased workload and poor workflow owing to poor implementation into existing clinical systems. Further research should investigate the implementation, cost-effectiveness, safety, and equity of asynchronous telemedicine use.

## Introduction

**Figure 1.**
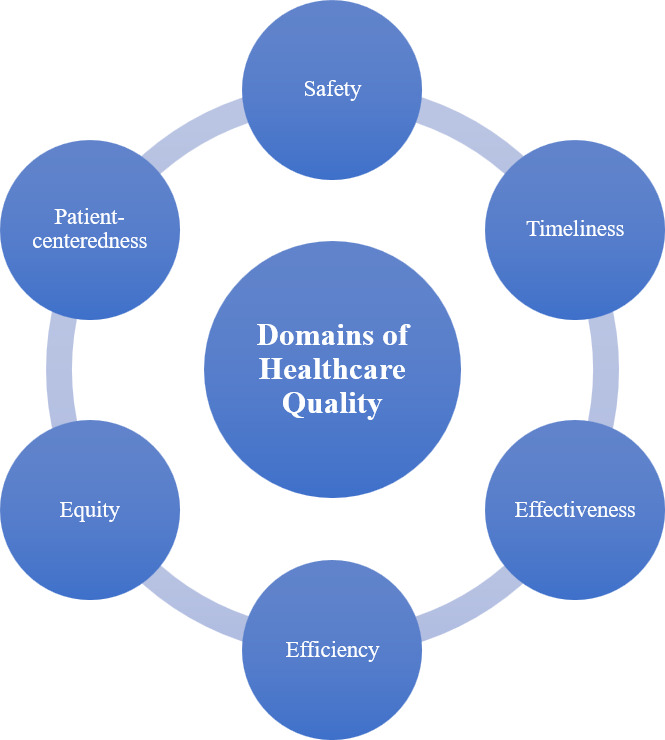
Domains of healthcare quality^3^

Telemedicine, the use of telecommunication for providing remote health assessments and therapeutic interventions, as defined in Box 1, has been used in health care for several years and there is a global focus on its development, owing to the rapid increase in use following the COVID-19 pandemic outbreak.^1^ During the pandemic, 99% of general practices in the UK adopted remote consultation platforms, which was a major change in practice and a move towards asynchronous telemedicine, allowing data to be transmitted, stored, and interpreted later.^2^ However, it is unclear whether asynchronous telemedicine allows healthcare professionals to provide quality care for patients according to the domains of healthcare quality: safety, timeliness, effectiveness, efficiency, equity, and patient-centredness, as outlined in Figure 1.^3^

Box 1 **Definitions of telemedicine, synchronous telemedicine, asynchronous telemedicine, and general practice**

- **Telemedicine:** '*The use of telecommunication and information technology for the purposes of providing remote health assessments and therapeutic interventions.*' NHS England^55^
- **Synchronous telemedicine:** '*Real-time, audio-video and telephone communication that connects physicians and patients in different locations.'* American Medical Association^6^
- **Asynchronous telemedicine:** Also known as the 'store-and-forward' technique. It allows data, including text and images from online services, to be transmitted and interpreted later.^6^
- **General practice:** General practice is the first point of contact for patients to access healthcare services. It offers a range of services, including consultations, prescriptions, treatments and management of long-term conditions, referrals to specialists, and health promotion. A wide range of practitioners work in general practice including doctors (GPs), nurses, and other allied health professionals.^56^

**Figure 2.**
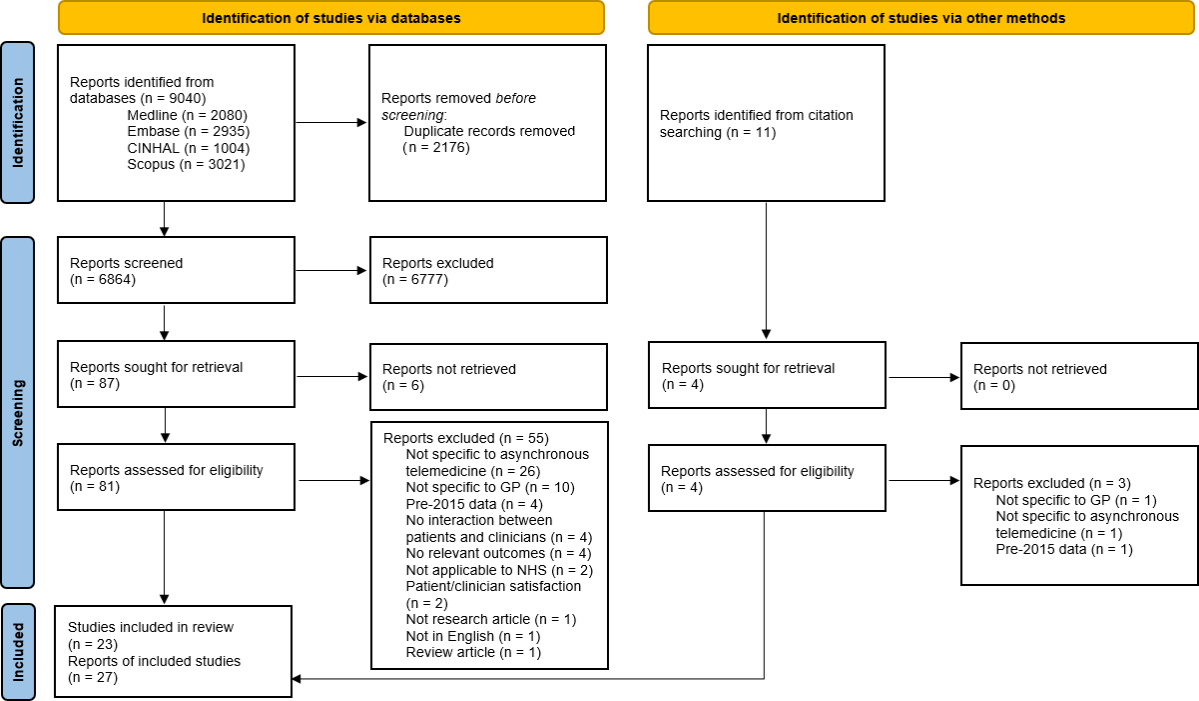
PRISMA flow diagram

Increased policy directives for telemedicine include the NHS Long Term Plan, aiming for ‘digital first’ primary care by 2023–2024 through the NHS App.^4^ The Welsh Government aims for remote delivery of 35% of initial and 50% of follow-up appointments.^5^

Asynchronous telemedicine occurs through secure messaging, such as texting and online platforms, and can involve clinical decision-making aids.^6,7^ Uses include: evaluating whether patients need further consultations; and communication between patients and healthcare professionals, or between multiple healthcare professionals.^6^ The focus of this review is consultations between patients and healthcare professionals for medical advice.

Synchronous telemedicine, which includes video and telephone consultations, has been more widely researched than asynchronous telemedicine.^8–10^ Existing reviews have focused on areas such as sharing images for dermatology consultations, and specific types of secure messaging such as emails.^11,12^ There is a recent rapid review on the value of asynchronous communication between patients and physicians in primary care,^13^ but none focusing specifically on quality of care. A pre-pandemic review on uses of e-consultations in primary care highlighted that research into effectiveness and safety of asynchronous telemedicine is needed.^14^

Asynchronous telemedicine has potential to change service delivery in the UK and internationally as 90% of NHS consultations occur in general practice,^15^ there are high levels of public interest in access to GP appointments,^16–18^ and practice has changed following COVID-19. Therefore, reviewing this field is important. Results will be guided by the domains of healthcare quality, a widely accepted model of healthcare quality,^3^ which will assist in identifying evidence gaps.

### Aim and objectives

The aim of this review was to investigate uses and effectiveness of asynchronous telemedicine in general practice. The specific objectives were to:

- identify types of asynchronous telemedicine used in general practice;
- assess how asynchronous telemedicine in general practice performs on each domain of healthcare quality;
- describe how asynchronous telemedicine use has changed since the COVID-19 pandemic outbreak in March 2020.

## Method

This study occurred between October 2022 and April 2023, and is reported according to the Preferred Reporting Items for Systematic Reviews and Meta-Analyses (PRISMA) guidelines.^19^

### Search strategy

Following pilot searching, MEDLINE, CINAHL, Embase, and Scopus were searched. Search themes included general practice, asynchronous telemedicine, uses, effectiveness, and safety (full details in Supplementary Figure S1). Further relevant studies were identified from reference lists of included reports.

Reports published between January 2015 and 31 November 2022 were searched to identify literature published before and following the COVID-19 pandemic outbreak in March 2020.

### Study selection

Eligibility criteria, detailed in Table 1, were developed using Population, Intervention, Comparison, Outcome (PICO) framework.^20^

**Table 1.** Inclusion and exclusion criteria

	Inclusion	Exclusion
Population	Patients and staff who have used asynchronous telemedicine in a general practice setting, including all healthcare professionals and other members of staff and consultations relating to all patient groups, including adults, children, and carers.	Other areas of primary care: dentistry, optometry, community nursing, pharmacy.Secondary and tertiary care.
Intervention	All methods of asynchronous telemedicine; for example, e-consults, secure messaging, text consultations, eVisits, emails.Interactions between patients and healthcare professionals seeking medical advice.	Synchronous telemedicine such as video appointments, telephone appointments.Automated asynchronous telemedicine, telemonitoring, interactions between two or more healthcare professionals.
Comparison	Face-to-face consultations.Synchronous telemedicine.No comparison.Before, and following the outbreak of the COVID-19 pandemic.	
Outcomes	Uses of asynchronous telemedicine.Safety Adverse events, harm caused or medical errors. Timeliness Time to appointment. Effectiveness Diagnosis made or resolution of problem. Treatments delivered, for example, prescribed medication. Number of appointments arranged and attended following asynchronous consultation and the type of follow-up. Efficiency Effect on workflow for healthcare professionals and patients, cost-effectiveness. Reduction or replacement of other types of consultations. Equitability Access for patients. Patient-centredness Perceptions of patients and healthcare professionals.	
Study design	Empirical research:Quantitative studies: comparative and observational studies.Mixed-methods studies.Qualitative studies.	Healthcare policies.Editorials and opinion pieces.Case studies.Study protocols.
Other	English language.Studies including data from 2015 onwards.Studies involving healthcare systems that are comparable with the NHS, for example, OECD countries.	

OECD = Organization for Economic Cooperation and Development

One researcher (CL) screened search results against inclusion criteria and 10% were independently screened for agreement (AP). One researcher (CL) screened full texts and queries were discussed within the research team. Search results and inclusion decisions were recorded using EndNote (version 20).^21^

### Data extraction

CL carried out data extraction. A template was piloted and discussed within the research team. The final template (Supplementary Figure S2) was based on this review’s objectives and assisted consistent data extraction across studies, including the design, participant characteristics (staff or patients), type of asynchronous telemedicine, and comparators. Also retrieved were each study’s objectives, patients involved, main findings, and whether the results were before or following the COVID-19 pandemic outbreak.

### Critical appraisal

Critical appraisal was at study level with the aid of the Mixed Methods Appraisal Tool (MMAT) for qualitative, quantitative, and mixed-methods studies.^22^ The Critical Appraisal Skills Programme (CASP) checklist was also used for qualitative studies.^23^ There was no formal risk of bias due to heterogeneity of included studies.

### Data synthesis

A narrative synthesis approach was used.^24^ Results were grouped and synthesised according to the study’s objectives and six domains of healthcare quality by one author (CL), and this was discussed within the research team until agreement was reached.^3^

No meta-analysis was carried out owing to inclusion of qualitative data.

## Results

### Search results

Database searching returned 9040 reports. After removing duplicates, 6864 remained; 6777 were excluded through title and abstract screening, so 87 remained. Eighty-one were retrieved and assessed for eligibility. Six were inaccessible. Eleven further records were retrieved through citation searching; four were assessed for inclusion following title and abstract screening, and three of these were excluded. Twenty-seven reports from 23 primary studies were included.

Figure 2 summarises screening and Supplementary Table S2 details reasons for exclusion at full text.

### Study characteristics

Studies were from the UK (*n* = 9), the US (*n* = 5), Spain (*n* = 2), Sweden (*n* = 1), Switzerland (*n* = 1), The Netherlands (*n* = 1), Republic of Ireland (*n* = 1), Norway (*n* = 1), Canada (*n* = 1), and Israel (*n* = 1).

Designs included the following (some studies used more than one design): interview studies (*n* = 8); cross-sectional surveys (*n* = 8); free-text responses (*n* = 3); cross-sectional data (*n* = 2); qualitative data (*n* = 2); and cohort studies (*n* = 10).

Three studies compared the COVID-19 pandemic period with the period before the pandemic,^25–28^ and one addressed only the pandemic period.^27^

Supplementary Table S1 details study characteristics.

### Types of asynchronous communication

Table 2 details asynchronous telemedicine types reported. This review focused on consultations for medical advice, but other uses included prescriptions, sickness certification notes, and managing appointments.

**Table 2.** Types of asynchronous communication reported

Online platforms (*n* = 21)	
eConsult, UK^26,31–33,40,45^	Online questionnaire initiated by patients with responses via email, text, or synchronous consultations (telephone or face-to-face).Responses next working day.
eConsulta, Spain^28,46,47^	Two-way online messaging between patient and clinician.Consultations initiated by patients or healthcare professionals.
Zipnosis, US^36,37^	Patient questionnaire, including free-text options, which is responded to by a physician with the aid of a decision support algorithm.Responses within 1 hour during business hours (8am–8pm).
Docly, UK^42^	Online questionnaire with included decision support algorithms and responses from GPs via secure messaging within the portal.
Digital dialogue with the GP, Norway^39^	Electronic consultation with GPs through online portal.Part of a wider online service that also offered non-clinical services to patients.
Other: secure messaging, websites, apps, eVisits or a mixture.	
**Email (*n* = 2**)^49,57^	
GPs give out personal or practice email address to patients.Some managed by administrative staff, some by GPs.	
**Text messages (*n* = 3**)^29,43,57^	
GPs give out personal or practice phone number to patients.Some managed by administrative staff, some by GPs.	

Some of the studies report on more than one mode of asynchronous telemedicine.

### Staff use of asynchronous telemedicine

GPs, nurses, and administrative staff use asynchronous telemedicine. One study reported female GPs were more likely to text patients,^29^ but one found no difference in use by healthcare professionals’ sex or age.^30^

### Patient use of asynchronous telemedicine

Patients from a range of demographics were included, consulting for new and ongoing concerns. Eighteen studies involved adult general practice patients. Six involved patients with predefined conditions only.

Studies reported 57–79% of asynchronous consultations were completed by female patients. Six reported patients who used asynchronous telemedicine were younger on average than users of face-to-face consultations,^31–39^ but use was reported across all age groups. Two studies found socioeconomic factors had no effect on uptake,^31–33,40^ whereas others reported differences in use between religious and ethnic groups,^25^ higher uptake from patients in rural areas,^28,41^ and higher uptake from patients with higher education levels.^39^

### Study quality

Studies had appropriate designs to address their aims. However, owing to many being observational, non-response bias was a limitation. This means their results cannot be assumed to be representative of study populations, which is important to consider when interpreting the results of this review, which offers a descriptive overview of existing evidence and suggests where gaps lie. More significant limitations include omissions in methodology, such as overlooking confounders or reasons for missing data. Qualitative studies lacked details of data saturation.

Supplementary Table S1 includes study specific comments and Supplementary Table S3 details critical appraisals.

### Domains of healthcare quality

#### Safety

Studies showed no differences between numbers of patients admitted to hospital or seeking emergency care according to consultation type; however, safety outcomes are not widely reported.

One study found 55.6% of GPs in Ireland obtain specific consent when texting medically sensitive information, 29.8% do sometimes, and 23.6% never do, raising consent and confidentiality concerns.^29^

To avoid receiving a ‘phone 999’ message when using eConsult, patients downplayed symptoms.^26^ It is unclear whether this problem with platform usability caused safety concerns. One example of a safety concern was a patient requiring further medication at follow-up; the authors inferred this meant their condition could have worsened following asynchronous consultation.^42^

#### Timeliness

Most asynchronous platforms were available 24 hours, 7 days a week. Some had expected response times, ranging from 15 minutes^34,35^ to 48 hours.^43^ Two studies found patients completing virtual consultations reported shorter symptom durations before consultation than those who had face-to-face consultations.^36,37^

#### Effectiveness

##### Diagnoses and investigations

One study found diagnoses were made based on symptoms following 25% of asynchronous consultations, compared with 14.2% of face-to-face consultations.^34,35^ Face-to-face consultations resulted in more investigations,^36^ but more inappropriate diagnoses.^37^

##### Prescriptions

One study found 58% of patients received a prescription following asynchronous consultation;^44^ for example, antibiotics, birth control, and respiratory medications. Antibiotic prescriptions were in line with guidelines more often following e-consultations than face-to-face consultations and fewer were prescribed following e-consultations.^34–37^

##### Resolution of queries and further appointments

Patient reported resolution of queries occurred in 33–66% of cases following asynchronous consultation. One study reported complete resolution more often following e-consultations than face-to-face consultations (55% versus 33%).^45^ Fewer patients felt able to provide all relevant information during e-consultation and resolution was not related to whether the consultation was initiated by the patient or clinician.^46,47^ It is unclear whether clinicians felt queries were resolved and what reasons existed for unresolved queries.

Follow-up rates ranged from 25.8–66.1%. One study found mean follow-up time was 1.2 days.^44^ Many were telephone or face-to-face, (55.3–74%), which were more likely when patients had new or complex problems,^31–33^ or required physical examination.^34,35^ Compliance with follow-up recommendations varied; 17.6–87.5%.

##### Healthcare professionals’ confidence

Clinicians’ experience with asynchronous telemedicine could influence outcomes.^31–33^ One study found GPs felt confident with 97% of requests received,^45^ but another reported some limited confidence.^48^

### Efficiency

#### Comparison with other consultation types

E-consultations were considered by GPs in one study as potentially being able to replace 55–88% of face-to-face consultations.^46,47^ Timewise, they take between 2.5 and 10 minutes, so are equivalent to telephone and face-to-face consultations.^30,40^

One study reported 21% of practices previously used electronic messaging but stopped; it is unclear why.^49^

#### Effects on staff workflow

Six studies reported asynchronous telemedicine added to clinical and administrative workload, through adding a stream of work and increasing demand, but it is unclear whether this is offset by reductions in other consultations.

One study reported e-consultations led to more screen time, less interaction with people but promoted teamwork.^48^ Barriers to improved workflow included poor communication, information flow, and usability of online systems.^50^ Positive effects were convenience for staff and saving time on administrative tasks and other consultations.

#### Patient non-adherence to systems

Eight studies reported patient non-adherence to asynchronous systems negatively affected workflow and staff thought patients used them as a ‘shortcut’ to other consultations.

#### Costs

Costs or savings would be influenced by the efficiency of systems, as this determines whether other consultation types have been replaced, potentially saving resources for practices and staff. An economic evaluation of eConsult found no added costs, but they were unable to tell if savings were made owing to low usage.^40^ Otherwise, costs were poorly reported.

### Equity

Equity is not widely reported. Qualitative evidence suggested asynchronous telemedicine could improve access to general practice for patients with hearing difficulties, and those who are housebound or have caring responsibilities.^29,48^ Concerns included digitally excluded patients and reinforcing health inequities.^31–33,43,48^

### Patient-centredness

Eight studies reported benefits for patients, including convenience, as asynchronous consultations can be completed out of hours and at home, saving an average of 1 hour in travel, waiting and consultation time, and travel costs. Patients reported faster responses and improved quality of treatment.^39^ One study reported asynchronous telemedicine promoted patient engagement and empowerment.^50^ Negative effects included increased responsibility for patients and laborious questionnaires.

### Changes following COVID-19 pandemic outbreak

Studies reported low asynchronous telemedicine use before the pandemic; up to seven consultations per 1000 patients per month.^31–33,35^ Three studies reported patient use of asynchronous telemedicine increased gradually before the pandemic^26,31–33,40^ and in 2016 20% of UK general practices planned to introduce it.^49^

Four studies reported use of asynchronous telemedicine increased rapidly following the COVID-19 pandemic outbreak in March 2020.^25,27,43,50^ One study found 70.88% of users avoided face-to-face primary care during the pandemic,^30^ with e-consultation rates increasing from 5.61 per 1000 patients in March 2020 to 33.1 per 1000 patients in June 2020 in one healthcare system.^28^ Despite increased use, only 32% of practices in The Netherlands intended to continue using e-consultations.^27^

Average asynchronous telemedicine users during the pandemic were younger, more likely to be employed. and had fewer chronic diseases than average users before the pandemic,^28^ and the gap between numbers of female and male users increased.^26^

Table 3 provides a synthesis of results and the gaps in the evidence base identified in this review.

**Table 3.** Summary of results and evidence gaps according to study objectives

Objective		Evidence	Gaps
Identify types of asynchronous telemedicine used in general practice.		Online platforms are most used. Text messages and email also used. Implementation differed between countries, platforms, and sometimes practices or individual clinicians. Used by a range of general practice staff. Use reported across all patient demographics. Used more by females and younger people.	Unclear why implementation differed between practices. Unclear whether groups of healthcare staff are more or less likely to use asynchronous telemedicine. Reasons for younger people and female patients using asynchronous telemedicine more than older people and male patients are needed. Unclear whether demographic factors, such as religion, ethnic group, socioeconomic status, and geographical location, affects use.
Assess how asynchronous telemedicine in general practice performs on each domain of healthcare quality.	Safety	No differences in numbers of patients admitted to hospital or seeking emergency care according to consultation type. Concerns surrounding consent and confidentiality.	Not widely reported in included studies. Studies using clear clinical end-point safety measures are required.
	Timeliness	Many platforms available 24 hours, 7 days a week, with clear response times up to 48 hours. Patients reported shorter symptom duration before asynchronous consultation.	Response times often poorly reported.
	Effectiveness	More accurate diagnoses made and fewer investigations. Range of prescriptions issued. Antibiotic prescriptions more often in line with guidelines. Patients reported resolution of queries in many cases, but fewer felt able to provide all relevant information. Many follow-ups were face-to-face or telephone. Many clinicians felt confident dealing with asynchronous consultations.	Unclear whether clinicians felt patient queries were resolved during asynchronous consultation. Reasons for unresolved queries are unclear. Owing to range in reported follow-up rates (25.8%–66.1%) we cannot know if other consultation rates (face-to-face or telephone) are being reduced.
	Efficiency	Two studies reported asynchronous telemedicine could replace more than half of face-to-face consultations. They take equivalent length of time to face-to-face and telephone consultations. Reports of additional workload for clinical and administrative staff, but also reports of time savings. Barriers to improved workflow: poor communication, lack of usability, and information flow. Patient non-adherence negatively affects workflow. One economic evaluation reported no added cost but unable to tell whether there are savings.	Unclear what type of consultations can and cannot be carried out asynchronously and reasons for this. Contradictory reports of increased workflow but also time savings for clinical and administrative staff. Unclear why there is a lack of usability and information flow, whether problems with the platform or its implementation. Reasons for patient non-adherence. Further economic evaluation is required.
	Equity	Qualitative evidence suggests improved access for some groups. Concerns regarding digitally excluded patients and reinforcing existing health inequities.	Not widely reported in included studies. Further studies are required to identify whether specific groups are excluded and advantaged or disadvantaged by using asynchronous telemedicine.
	Patient-centredness	Reports of benefits: convenience, savings in travel time and costs, faster treatment. Increased engagement and empowerment. Reports of questionnaires being laborious.	Patient involvement in design of platforms to ensure usability clear.
Describe how asynchronous telemedicine use has changed since the COVID-19 pandemic outbreak in March 2020.		Huge increase in use of asynchronous telemedicine from March 2020. Allowed face-to-face consultations to be avoided. Users were younger, employed, with fewer chronic conditions, and more female patients.	Unclear whether increased use has been maintained. Reasons for younger people and female patients using asynchronous telemedicine more are needed.

## Discussion

### Summary

Asynchronous telemedicine is used by a range of staff and patients worldwide. It can be effective in making diagnoses, prescribing medications, and takes equivalent time to face-to-face and telephone consultations. For patients, it can provide timely access to general practice and save on travel time and costs. Hindrances to efficiency are reported, such as increased clinical and administrative workload and barriers to workflow, such as poor usability. Safety and equity are poorly reported, but concerns include consent, confidentiality, and reinforcing health inequalities. Its use increased rapidly following the pandemic outbreak in March 2020.

### Comparison with existing literature

A pre-pandemic review of e-consultations in primary care found similar patterns of patient use, including more female users and use across all age groups. Mold and colleagues found socioeconomically disadvantaged patients used e-consultations less, which is inconsistent with our findings.^14^ However, there is a lack of causative evidence for this, as our findings in this domain are from qualitative sources.

Consistencies with synchronous telemedicine include providing time and cost savings for patients,^51–53^ and evidence suggesting a lack of guidance for use of both makes confidentiality a concern.^14,54^ Synchronous telemedicine can provide cost savings for healthcare systems,^11^ but costs are poorly reported for asynchronous telemedicine.

Mold *et al* found (i) patients were more likely to use e-consultations if they thought a face-to-face consultation was not needed, and (ii) no increased workload for clinicians, both of which findings are inconsistent with our findings.^14^ These differences could be influenced by their review, including synchronous and asynchronous telemedicine and being carried out before COVID-19, or could be owing to the qualitative nature of evidence found in our review.

### Strengths and limitations

A limitation is that data extraction and synthesis were carried out by only one researcher. To minimise this, both processes were discussed within the research team throughout, until agreement was reached.

There was no formal risk of bias assessment owing to the heterogeneity of included studies. However, studies were critically appraised using the MMAT and additionally CASP for qualitative studies, which is a strength.

All included studies were observational or qualitative and many were on a small scale, which limits the clinical significance of their results. Additionally, the heterogeneity between types of asynchronous communication reported makes it difficult to compare studies. This limits how definitive the findings of this review can be, which is why a narrative synthesis approach was chosen, allowing a descriptive overview of the existing literature, and identification of significant evidence gaps.

A strength of this review is the use of the domains of healthcare quality,^3^ which are a globally recognised framework, and allow for the finding to be applied to worldwide healthcare systems. This is strengthened further by the inclusion of international literature.

### Implications for research and practice

Policymakers should focus on how to address ethical issues, such as documenting consent and patient information, to ensure awareness and manage expectations of asynchronous telemedicine in a safe manner.

A standardised approach to asynchronous telemedicine in general practice, such as introducing one platform, and defining enquiries it should be used for would improve practice and increase sustainability. This is important in the NHS as UK-based studies reported patient non-adherence to asynchronous systems.^26,30–33,40,43,45^

A standardised approach will be influenced by further research into the implementation of asynchronous telemedicine in general practice. This is of importance as there were reports of practices stopping using asynchronous telemedicine,^27,49^ problems with workflow, and increased workload despite reports suggesting it can replace face-to-face consultations, which could all be influenced by its implementation. Further, the COVID-19 pandemic offered a unique opportunity for asynchronous telemedicine to be studied as there was huge widespread implementation, and it is unclear whether this has been maintained.

Future research should address the safety, economic costs, time savings, and whether specific groups are advantaged or disadvantaged by using asynchronous telemedicine. This should be through high quality large-scale studies, such as randomised control trials and observational or cross-sectional studies, using clear clinical end-point outcomes.

In conclusion, asynchronous telemedicine, such as online platforms, text, and email, is used in general practice worldwide by many staff and patients. It can provide effective, efficient, and timely care, and benefits for patients. Increased workload for staff and barriers to efficient workflow are reported. The COVID-19 pandemic led to rapid increases in asynchronous telemedicine use. Further evaluation of cost-effectiveness, equity, and safety of asynchronous telemedicine is required, and studies of its implementation will inform future policy and enable sustainable practice.

## Trial registration number

The protocol was registered with PROSPERO; registration number CRD42023394484.

## Data

The dataset relied on in this article is fully avaliable in the results and supplementary materials.

## References

[1] Greiwe J (2022). Telemedicine lessons learned during the COVID-19 pandemic. Curr Allergy Asthma Rep.

[2] Hutchings R (2020). The impact of COVID-19 on the use of digital technology in the NHS.

[3] Institute of Medicine (US) Committee on Quality of Health Care in America (2001). Crossing the quality chasm: a new health system for the 21st century.

[4] NHS England (2019). NHS long term plan.

[5] Welsh Government (2022). Our programme for transforming and modernising planned care and reducing waiting lists in Wales.

[6] American Medical Association (2021). Telehealth resource center: definitions.

[7] Elliott T, Yopes MC (2019). Direct-to-consumer telemedicine. J Allergy Clin Immunol Pract.

[8] Edwards D, Csontos J, Gillen L (2022). A rapid review of the effectiveness of remote consultations versus face-to-face consultations in secondary care surgical outpatient settings [Preprint]. medRxiv.

[9] West HJ, Barzi A, Wong D (2022). Telemedicine in cancer care beyond the COVID-19 pandemic: oncology 2.0?. Curr Oncol Rep.

[10] Farrell A, George N, Amado S, Wozniak J (2022). A systematic review of the literature on telepsychiatry for bipolar disorder. Brain Behav.

[11] Deshpande A, Khoja S, Lorca J (2009). Asynchronous telehealth: a scoping review of analytic studies. Open Med.

[12] Goyder C, Atherton H, Car M (2015). Email for clinical communication between healthcare professionals. Cochrane Database Syst Rev.

[13] Fuster-Casanovas A, Vidal-Alaball J (2022). Asynchronous remote communication as a tool for care management in primary care: a rapid review of the literature. Int J Integr Care.

[14] Mold F, Hendy J, Lai Y-L, de Lusignan S (2019). Electronic consultation in primary care between providers and patients: systematic review. JMIR Med Inform.

[15] Goodwin N, Dixon A, Poole T, Raleigh V (2011). Improving the quality of care in general practice.

[16] Oliver D (2022). David Oliver: Facts the Daily Mail omits in its GP bashing. BMJ.

[17] Davis N (2020). Fear of contacting GPs during Covid outbreak “fueling missed diagnoses.”. The Guardian.

[18] Rees J (2021). GP appointments: Patients 'compete' when phones open. BBC News.

[19] PRISMA (2020). Preferred Reporting Items for Systematic Reviews and Meta-Analyses.

[20] Higgins JPT, Thomas J, Chandler J (2023). Cochrane Handbook for systematic reviews of interventions version 64.

[21] The EndNote Team (2013). Endnote 20.

[22] Hong QN, Fàbregues S, Bartlett G (2018). Mixed methods appraisal tool (MMAT) version 2018 for information professionals and researchers. EFI.

[23] Critical Appraisal Skills Programme (2022). CASP checklists.

[24] Centre for Reviews and Dissemination (CRD) (2009). Systematic reviews: CRD’s guidance for undertaking reviews in health care.

[25] Miron O, Wolff Sagy Y, Yaron S (2022). Trends in the volume and types of primary care visits during the two years of the COVID-19 pandemic in Israel. Int J Environ Res Public Health.

[26] Jones RB, Tredinnick-Rowe J, Baines R (2022). Use and usability of GP online services: a mixed-methods sequential study, before and during the COVID-19 pandemic, based on qualitative interviews, analysis of routine eConsult usage and feedback data, and assessment of GP Websites in Devon and Cornwall, England. BMJ Open.

[27] Keuper J, Batenburg R, Verheij R, van Tuyl L (2021). Use of E-health in Dutch general practice during the COVID-19 pandemic. Int J Environ Res Public Health.

[28] Solans O, Vidal-Alaball J, Roig Cabo P (2021). Characteristics of citizens and their use of teleconsultations in primary care in the Catalan public health system before and during the COVID-19 pandemic: retrospective descriptive cross-sectional study. J Med Internet Res.

[29] Quinlan D, Dahm M, Lyons A, Collins C (2018). Patient texting in general practice: who, why, why not? A national survey of text messaging in Irish general practice. Ir Med J.

[30] Casey M, Shaw S, Swinglehurst D (2017). Experiences with online consultation systems in primary care: case study of one early adopter site. Br J Gen Pract.

[31] Banks J, Farr M, Salisbury C (2018). Use of an electronic consultation system in primary care: a qualitative interview study. Br J Gen Pract.

[32] Edwards HB, Marques E, Hollingworth W (2017). Use of a primary care online consultation system, by whom, when and why: evaluation of a pilot observational study in 36 general practices in South West England. BMJ Open.

[33] Farr M, Banks J, Edwards HB (2018). Implementing online consultations in primary care: a mixed-method evaluation extending normalisation process theory through service co-production. BMJ Open.

[34] Entezarjou A, Calling S, Bhattacharyya T (2021). Antibiotic prescription rates after eVisits versus office visits in primary care: observational study. JMIR Med Inform.

[35] Entezarjou A, Sjöbeck M, Midlöv P (2022). Health care utilization following 'digi-physical' assessment compared to physical assessment for infectious symptoms in primary care. BMC Prim Care.

[36] Johnson KL, Dumkow LE, Salvati LA (2021). Comparison of diagnosis and prescribing practices between virtual visits and office visits for adults diagnosed with uncomplicated urinary tract infections within a primary care network. Infect Control Hosp Epidemiol.

[37] Johnson KM, Dumkow LE, Burns KW (2019). Comparison of diagnosis and prescribing practices between virtual visits and office visits for adults diagnosed with sinusitis within a primary care network. Open Forum Infect Dis.

[38] Stamenova V, Agarwal P, Kelley L (2020). Uptake and patient and provider communication modality preferences of virtual visits in primary care: a retrospective cohort study in Canada. BMJ Open.

[39] Zanaboni P, Fagerlund AJ (2020). Patients' use and experiences with E-consultation and other digital health services with their general practitioner in Norway: results from an online survey. BMJ Open.

[40] Cowie J, Calveley E, Bowers G, Bowers J (2018). Evaluation of a digital consultation and self-care advice tool in primary care: a multi-methods study. Int J Environ Res Public Health.

[41] Powell KR, Deroche C (2020). Predictors and patterns of portal use in patients with multiple chronic conditions. Chronic Illn.

[42] Nijhof D, Ingram A, Ochieng R (2021). Examining GP online consultation in a primary care setting in East Midlands, UK. BMC Health Serv Res.

[43] Murphy M, Scott LJ, Salisbury C (2021). Implementation of remote consulting in UK primary care following the COVID-19 pandemic: a mixed-methods longitudinal study. Br J Gen Pract.

[44] Penza KS, Murray MA, Pecina JL (2018). Electronic visits for minor acute illnesses: analysis of patient demographics, prescription rates, and follow-up care within an asynchronous text-based online visit. Telemed J E Health.

[45] Carter M, Fletcher E, Sansom A (2018). Feasibility, acceptability and effectiveness of an online alternative to face-to-face consultation in general practice: a mixed-methods study of webGP in six Devon practices. BMJ Open.

[46] López Seguí F, Vidal-Alaball J, Sagarra Castro M (2020). General practitioners' perceptions of whether teleconsultations reduce the number of face-to-face visits in the Catalan public primary care system: retrospective cross-sectional study. J Med Internet Res.

[47] López Seguí F, Walsh S, Solans O (2020). Teleconsultation between patients and health care professionals in the Catalan primary care service: message annotation analysis in a retrospective cross-sectional study. J Med Internet Res.

[48] Turner A, Morris R, Rakhra D (2022). Unintended consequences of online consultations: a qualitative study in UK primary care. Br J Gen Pract.

[49] Brant H, Atherton H, Ziebland S (2016). Using alternatives to face-to-face consultations: a survey of prevalence and attitudes in general practice. Br J Gen Pract.

[50] Hoonakker PLT, Carayon P, Cartmill RS (2017). The impact of secure messaging on workflow in primary care: results of a multiple-case, multiple-method study. Int J Med Inform.

[51] Michaud TL, Zhou J, McCarthy MA (2018). Costs of home-based telemedicine programs: a systematic review. Int J Technol Assess Health Care.

[52] Batsis JA, DiMilia PR, Seo LM (2019). Effectiveness of ambulatory telemedicine care in older adults: a systematic review. J Am Geriatr Soc.

[53] Mechanic OJ, Persaud Y, Kimball AB (2022). Telehealth systems.

[54] Scott Kruse C, Karem P, Shifflett K (2018). Evaluating barriers to adopting telemedicine worldwide: a systematic review. J Telemed Telecare.

[55] NHS England (2022). Telemedicine.

[56] Gregory S (2009). General practice in England: an overview.

[57] Dash J, Haller DM, Sommer J, Junod Perron N (2016). Use of email, cell phone and text message between patients and primary-care physicians: cross-sectional study in a French-speaking part of Switzerland. BMC Health Serv Res.

